# Liverpool Royal Infirmary

**Published:** 1917-05-26

**Authors:** 


### Liverpool Royal Infirmary.

We have received from Miss Margaret Cummins, the matron of the Royal Infirmary, Liverpool, the following sets of senior nurses' examination papers in special subjects, set at the April examinations, which are just over. These nurses have already attended lectures and passed examinations in elementary anatomy and physiology, medical nursing, gynæcological nursing, and the matron's course, and they are now doing sick-room cookery. Each specialist lectures, sets the questions, examines and marks the nurses' papers.

Senior Nurses' Examinations in Special Subjects.

Mr. Bickerton (Ophthalmology).—Questions: (1) Mention Nature's provisions for the protection of the eye. (2) Give causes and symptoms of (*a*) conjunctivitis, (*b*) corneal ulceration, and the treatment—local and constitutional—for each.

Mr. Blair Bell (Gynæcology).—Questions: (1) Describe the abnormal discharges that may occur from the vagina, and indicate their significance. (2) What are the positions adopted (*a*) for examination, (*b*) after operation, and what is the object of each? (3) What are the common causes of shock after an abdominal operation; how can they be avoided, and what methods are employed for the treatment of this condition?

Mr. Guthrie (Diseases of Throat, Nose, and Ear).—Questions: (1) What are the most common causes and results of nasal obstruction? (2) What are the chief dangers after operations on the throat, and how may they be avoided?

Dr. Leslie Roberts (Skin Diseases).—Questions: (1) What benefits result from hardness of cuticle? (2) When the surface becomes soft and flabby, what disease is likely to ensue? (3) What is the part of the follicle specially prone to lead to disease? (4) How is disease brought about by this part? (5) How does the sarcoptes scabiei obtain its food? (6) How do the pediculi obtain their food? (7) What diseases may be conveyed by pediculi?

Mr. Marsden (Materia Medica and Pharmacy).—Questions: (1) Define filtration, maceration, infusion, and decoction. Show how these processes are conducted, and give any examples. (2) Write a short account of the weights and measures. (3) Mention the chief anæsthetics and their special uses. Give the preparations of opium, belladonna, and Indian hemp, with their doses as far as you can. (4) What is the meaning of the terms "mixture," "drops," "ointment," "plaster," "lotion," "liniment," "pills," and "tablets"? Mention any of the antiseptic lotions with which you may be familiar, and any conveniences for making them.

**We should welcome copies of Examination Papers and Results from other matrons of training-schools.**

